# Combining Direct and Indirect Measurements to Assess Patients’ Satisfaction with the Quality of Public Health Services in Romania: Uncovering Structural Mechanisms and Their Implications

**DOI:** 10.3390/ijerph17010152

**Published:** 2019-12-24

**Authors:** Elena Druică, Viorel Mihăilă, Marin Burcea, Vasile Cepoi

**Affiliations:** 1 Centre for Research in Applied Behavioural Economics, Faculty of Business and Administration, University of Bucharest, 030018 Bucharest, Romania; elena.druica@faa.unibuc.ro (E.D.); viorel.mihaila@faa.unibuc.ro (V.M.); 2 Faculty of Business and Administration, University of Bucharest; 030018 Bucharest, Romania; marin.burcea@faa.unibuc.ro; 3 The Romanian Authority for Quality Assurance in Healthcare, 060022 Bucharest, Romania

**Keywords:** patients’ satisfaction, health services quality, PLS–PM modeling, mediation analysis

## Abstract

**Introduction:** Patients’ satisfaction was extensively researched over the last decades, given its role in building loyalty, compliance to treatment, prevention, and eventually higher levels of wellbeing and improved health status. Patients’ feedback on the perceived quality of health services can be incorporated into practice; therefore, understanding factors and mechanisms responsible for patients’ satisfaction allows providers to tailor targeted interventions. **Method:** A questionnaire assessing patients’ perception of the quality of health services was administered to a country-representative sample of 1500 Romanian patients. Using a partial least squares—path modeling approach (PLS-PM), with cross-sectional data, we developed a variance-based structural model, emphasizing the mediating role of trust and satisfaction with various categories of health services. **Results:** We confirmed the mediating role of trust in shaping the relationship between the procedural accuracy of health professionals, along with the perceived intensity of their interaction with patients, and patients’ experienced quality of the health services. We confirmed the mediating role of satisfaction by the categories of services in the relationship between waiting time on the premises, attention received, and the perceived reliability of the information received, as predictors, and the experienced quality of the health services. In addition, indirect assessment of patients’ satisfaction is a good predictor for direct assessment, thereby affirming the idea that the results of the two types of evaluations converge. **Discussions:** One of the most efficient solutions to increase both patients’ satisfaction and their compliance is to empower the communication dimension between patients and health practitioners. Given the non-linear relationships among variables, we advocate that, unless the nature of the relationships between satisfaction and its predictors is understood, practical interventions could fail. The most relevant variable for intervention is the degree of attention patients perceive they received. We suggest three methods to turn waiting time into attention given to patients.

## 1. Introduction

Patients’ satisfaction was extensively researched over the last decades, with various systematic reviews encompassing the most relevant studies in the field [1,2,3,4]. Satisfaction prompts loyalty, compliance to treatment, and prevention [5], and it eventually translates into higher levels of wellbeing, lower levels of out-of-pocket expenditures to manage unexpected health events, and improved health status and happiness [6].

Understanding factors and mechanisms responsible for patients’ satisfaction allows providers to tailor targeted interventions [7] and helps health practitioners to improve their approach [8,9,10]. A prevalent research area is assessing patients’ satisfaction with separate categories of practitioners: doctors [11,12], nurses [13], and doctors and nurses [14], as well as with various categories of services or healthcare centers [15]. In this paper, we aim to explore the mechanisms that shape patients’ satisfaction, as well as patients’ self-perception of quality, taking into account their interaction with three categories of health professionals: doctors, nurses, and hospital housekeepers.

Our contribution to the existing literature is multi-fold. Firstly, we explore, empirically, how patients’ actual experience with health services shapes their perception of the quality of the health services. Unlike most of the previous studies, we combine direct and indirect assessments [2]. We investigate how satisfied patients are with the services received, which is a direct assessment, but we also ask the patients to rate different aspects of their experience, which is an indirect assessment of their satisfaction.

In explaining how satisfaction and perceived quality of the health services are shaped, we combine predictors coming from two different theoretical backgrounds: human capital and social capital. On the one hand, we place waiting time on the premises, attention given to patients, and patients’ trust in the information received from health professionals as dimensions related to human capital. On the other hand, we place health professionals’ perceived procedural accuracy and support provided in their interaction with patients as part of social capital.

Then, we look into potential mechanisms underlying the relationships between our predictors and the perceived quality and identify two relevant mediators. Other studies also approached research on patients’ satisfaction using structural modeling [16,17,18,19,20,21]. Based on the results, we inform practical interventions.

The rest of the paper is organized as follows: the next section presents the conceptual model and the literature review. Section 3 introduces the data, the measurement tools, and the method. Section 4 presents the results, while the last section concludes our research, suggests theoretical and practical implications, and presents the limitations of our work.

## 2. Background

**Figure 1:**
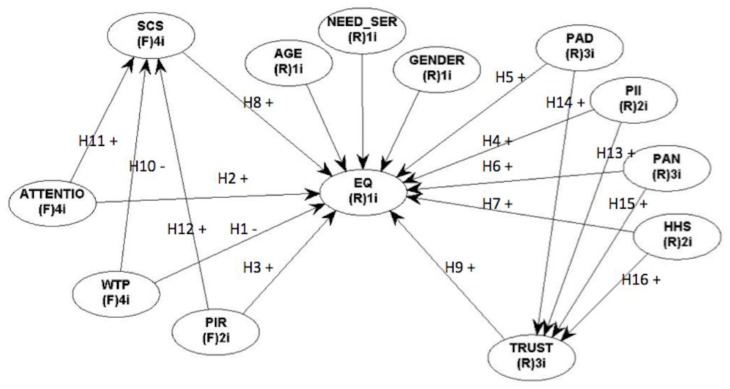
Conceptual research model and hypotheses.

Our primary goal, the importance of which is supported in recent literature [22], is to identify the determinants of patients’ perception of the experienced quality of health services (henceforth EQ), which are relevant for practical interventions. We focus on patients’ perceived experience with four types of health institutions: family physicians, specialists, hospitals, and laboratories. Firstly, we examine how the patients’ perception of their experience and the health practitioners’ attitude toward them [23] affect satisfaction with specific services, while also investigating their level of trust. Secondly, we discuss how all these factors impact EQ. Figure 1 shows our conceptual model, along with the research hypotheses, while Table 1 summarizes the main acronyms of the variables. The following subsections discuss the role played by each variable included in our study and ground the research model in the existing literature. We explain the mediating role of two latent variables as mechanisms that can explain the relationship between the EQ and its predictors.

### 2.1. Waiting Time, Attention, and Information Reliability

Waiting time on the premises (henceforth WTP), and attention received by patients in their interaction with health professionals (henceforth ATT) are both documented in the literature as building satisfaction [24,25]. Apart from that, lay-people experience information asymmetrically when relating to health services. Although they need the health practitioner to provide reliable information and psychological comfort, patients are increasingly skeptical about expert opinions, want more autonomy, and are eager to choose between different options presented by the health professionals [26]. The perceived quality of the actual interaction of patients with their doctor influences both the satisfaction and the level of concern about one’s health. The quality of this interaction is assessed differently by the doctor and the patient; what the patient views as important may be different from what the physician thought was important. We set perceived information reliability (henceforth PIR) as the predictor and set the first three hypotheses as follows:

**Hypothesis** **1** **(H1).** *WTP is negatively correlated with EQ.*

**Hypothesis** **2** **(H2).** *ATT is positively correlated with EQ.*

**Hypothesis** **3** **(H3).** *PIR is positively correlated with EQ.*

### 2.2. Perceived Interaction and Health Professionals’ Procedural Accuracy

Social capital covers the level of civic participation, trust, and social networks existing within specific communities. Social capital can be regarded as a property of an individual providing access to different resources [27], or Putnam’s five-dimensions perspective [28,29], or a group perspective [30]. Regardless of its conceptualization, social capital is responsible for the perception of quality in health services [31].

In this context, we refer to social capital as the relationship between health practitioners and patients [32]. Using McKenzie’s three-dimensional model, we measured patients’ perception toward professionals’ procedural accuracy (henceforth PAD for medical doctors; PAN for medical nurses) and support (HHS for hospital housekeepers), as reflected in their interpersonal relationships. These are all documented as predictors of satisfaction and perceived quality [33,34]. McKenzie’s model was chosen because it includes a structural and cognitive dimension, a bonding and bridging dimension, and a horizontal and vertical dimension, which we found to be the best fit for our conceptualization. We also measured the extent to which patients valued the information exchange with these professionals, concerning their intimate issues, medication, and treatment [35], and we labeled this variable “perceived intensity of interaction” (henceforth PII). We build our argument on the idea that, in the first instance, information asymmetry exists between the patient and health professional. The gap decreases through communication, in turn developing the patient’s confidence [36,37] and the perception of better control [38]. In this context, the following four hypotheses were proposed:

**Hypothesis** **4** **(H4).** *PII is positively correlated with EQ.*

**Hypothesis** **5** **(H5).** *PAD is positively correlated with EQ.*

**Hypothesis** **6** **(H6).** *PAN is positively correlated with EQ.*

**Hypothesis** **7** **(H7).** *HHS is positively correlated with EQ.*

### 2.3. The Mediating Effect of Satisfaction by Category of Services and Trust

The ability to find answers to patients’ problems provides the cornerstone to developing trust in professionals’ expertise and experience. It also makes patients see the health professionals’ recommendations and information as reliable, which, in turn, plays a crucial role in patients’ assessment of service quality and results in satisfaction [39,40,41]. In our paper, we measured patients’ satisfaction by the category of services (henceforth SCS), referring, in particular, to family physicians, specialists, hospital services, and laboratories. The process of value creation in vulnerable customers is important [42], and previous research showed that competent delivery of professional health services develops trust (henceforth TRUST) in patients [43]. As a result, the final nine hypotheses were as follows:

**Hypothesis** **8** **(H8).** *SCS is positively correlated with EQ.*

**Hypothesis** **9** **(H9).** *TRUST is positively correlated with EQ.*

**Hypothesis** **10** **(H10).** *WTP is negatively correlated with SCS.*

**Hypothesis** **11** **(H11).** *ATT is positively correlated with SCS.*

**Hypothesis** **12** **(H12).** *PIR is positively correlated with SCS.*

**Hypothesis** **13** **(H13).** *PII is positively correlated with TRUST.*

**Hypothesis** **14** **(H14).** *PAD is positively correlated with TRUST.*

**Hypothesis** **15** **(H15).** *PAN is positively correlated with TRUST.*

**Hypothesis** **16** **(H16).** *HHS is positively correlated with TRUST.*

### 2.4. Control Variables

There is conflicting evidence regarding gender and age as predictors of patients’ satisfaction and their experience with the quality of healthcare services. Some studies found that older patients tend to be more satisfied [44,45], but the results were context-dependent. Similarly, there is no consensus about possible gender differences, although some studies reported men as slightly more satisfied than women [46].

Measuring patients’ experience is also sensitive to the moment of measurement. If an experience is not measured at the very moment of its end, then memory self-evaluation and experience self-evaluation compete for how the experience is recalled [47]. In line with Kahneman et al.’s findings [41], other studies analyzing the patients’ account of their last consultation also documented the experienced utility and the remembered utility [48]. Differentiating between patients’ perception and patients’ experiences with medical care is of paramount importance; while the patients’ perception deals with expectations and a subjective check against reality (what was actually happening), the patients’ experiences are merely a kind of reflection of what happened to them and if their needs were met. Sometimes patients might overrate satisfaction due to different biases.

People adopt general categories to organize their memories about other people or experiences in social contexts. The individual acts in a socially determined framework based on their expectations about a particular situation [49]. Although effective most of the time, this strategy has the potential to generate false memories, because sometimes people remember category-consistent information that never occurred in that context [50]. To control the potential effects of assessing the respondents’ experience after it occurred, we included a dummy variable indicating whether or not the respondents had contact with medical services in the last 12 months.

## 3. Data, Measurement, and Method

### 3.1. Data

We collected data through a questionnaire comprising 27 questions aimed at assessing 10 different dimensions of the perceived quality (henceforth PQ) of the Romanian health services. The questionnaire, available in the Supplementary Materials, followed the logic of the conceptual model presented in Figure 1, and previous studies provided valuable guidance in developing the items included in Table 1 [51,52,53,54]. The original questionnaire was developed in the Romanian language and discussed with representatives of the Romanian Authority for Quality Management in Healthcare, for content validation. Then, a pilot study conducted with 30 respondents confirmed that the questions were articulately phrased.

### 3.2. Imputation

Among the 1500 respondents, some did not respond to several questions. The number of missing answers varied by question, from 11 to 250. Handling missing data is a common problem in social sciences. The literature addresses this challenge in three ways: deleting observations with missing entries and working with the complete cases only, weighting, and imputation [55]. In this research, we used the first and third strategies. For the third strategy, we implemented two types of imputation: arithmetic mean and multiple regression imputation [56,57]. To account for potential biases, we compared the estimation obtained in the “complete case” condition, with the estimations obtained in the two “imputation” conditions. We found no significant differences in our results.

### 3.3. Measurement

We looked at the degree of satisfaction in patients using two approaches. To measure overall satisfaction indirectly, we asked the respondents to rate their first-hand EQ of the Romanian public health services. Then, we asked them to self-assess their satisfaction by category of services provided by family physicians, specialists, hospital services, and laboratories. The measurement was on a 1–5 Likert scale (1—“totally unsatisfied”, 5—“very satisfied”). The latent predictors involved in our analysis were measured based on the items presented in Table 1. The items mirror similar measures used in previous studies [35,58,59,60]; however, in this particular form, they are our contributions.

### 3.4. Method

The partial least squares-path modeling approach (PLS–PM) technique [61,62,63,64,65,66] was used to explore the mediation effect of TRUST and SCS on EQ. The PLS–PM analysis performed in our paper aimed at estimating theoretically established relationships, by maximizing the explained variance of the dependent, endogenous latent variables, with EQ as the primary explained variable, and SCS and TRUST serving as mediators in our case. We found this method appropriate for testing our model as it is preferred whenever the theoretical background is insufficient, the measures do not conform to a specific model, and the variables do not fit a certain distribution [67]. A detailed description of the advantages of this method can be found in Reference [68].

The estimation method is an iterative algorithm based on ordinary least squares. Any PLS–PM model consists of two parts: an outer or measurement model and an inner or structural model. The outer model assesses the relationships of the latent constructs with their respective indicator manifest variables in terms of composite indices, while the inner model estimates the relationships among the latent variables themselves. The results of each stage are discussed in the subsequent sub-sections. We preliminary explored our data using R software version 3.4.3 (R Foundation for Statistical Computing, Viena, Austria), with the “plspm” package and the “plsdepot” package; then, we estimated our final models using WarpPLS version 6.0 software (http://www.warppls.com). The statistical inference of the results was based on a bootstrapping procedure with 999 repetitions.

The algorithm works with standardized data, namely, data transformed in such a way that each indicator has a mean zero and a standard deviation of 1, and it is able to capture linear and non-linear relationships among variables. In handling non-linear relationships, for each set of latent variables LV_1_, LV_2_, …, LV_k_, WarpPLS identifies a set of functions, F_1_, F_2_, …, F_k_ and a set of coefficients p_1_, p_2_, …, p_k_ such that a concept latent variable LVc (the outcome) can be expressed as LVc = p_1_ × F_1_(LV_1_) + p_2_ × F_2_(LV_2_) + … + P_k_ × F_k_(LV_k_) + E. Here, the *p*-values are path coefficients, and E is the error term. Depending on the estimation algorithm implemented for the inner model, the functions F_1_, F_2_, …, F_k_ can take U shapes (in Warp2 mode) or S shapes (in Warp3 mode) [69].

## 4. Results

Our final sample consisted of 1500 respondents (see Table 2 for descriptive statistics) representative of the Romanian population. The sampling was probabilistic, random, and stratified (regional, county level, and village/city level). We used a paper-and-pen approach, and the Romanian Center for Urban and Regional Sociology collected our data.

### 4.1. Measurement Stage (Outer Model)

The performance of the measurement was assessed using Cronbach’s alpha, a measure of internal consistency showing how closely related the manifest variables are in a specific group, the composite reliability index, showing the amount of total true score variance capture in a latent construct out of the total variance of the scale, and average variance extracted (AVE), showing how much variance is captured by a construct in relation to the variance due to the measurement errors. Our latent variables were suitable for measurement, as evidenced by the actual and the recommended values for each reliability index (Table 3). The only exception was WTP, for which Cronbach’s alpha and the average variance extracted were below the thresholds. Given the small number of items involved in this latent variable, we relied on the theoretical recommendation [70] and kept it in the analysis.

After applying a reflective measurement, we found that the manifest variables loaded into their corresponding latent constructs with at least 0.7 (Table S1) and were statistically significant. The only exception was for the latent variable WTP, for which the manifest items “waiting time to family doctors” and “waiting time to laboratories” showed loadings below 0.7. Despite the minor non-conformity in the loadings, these items were still statistically significant; thus, we kept them in the analysis. This stage confirmed the convergent validity of our measurement, showing that the items belonging to a specific construct were in fact related to that construct.

For discriminant validity, we found that the correlations of the latent variables were high (Table 4). In addition, all the diagonal values were higher than the corresponding off-diagonal values, and none of the off-diagonal values were higher than 0.8 [71]. This result shows that the constructs did not share the same type of items and that they were conceptually distinct.

### 4.2. The Inner (Structural) Model

Table 5 presents the coefficients of the estimated model. On the one hand, we discuss the total effect of each predictor on the perception of the overall quality; then, we deconstruct each total effect in terms of sum of direct effects and indirect effects, via mediators. On the other hand, we discuss the results in terms of effect sizes. This is important for managerial implications, as not all the statistically significant predictors are suitable for interventions, but only those with effect sizes beyond a certain threshold.

The *R^2^* values reported in Table 5 indicate a good explanatory power; our structural model explained 34% of the variations in how patients perceive the overall quality of the Romanian health services, and more than 30% of the variation in each mediator. For total effects, we found that PAN was the only category that did not affect EQ. The rest of the predictors were statistically significant.

WTP was negatively correlated with EQ which confirmed hypothesis H1. ATT was positively correlated with EQ; thus, H2 was accepted. For total effect, PIR was positively correlated with EQ; thus, H3 was confirmed. PII, PAD, and HHS were positively correlated with EQ, thereby confirming H4, H5, and H7. Although PAN held a positive coefficient, the corresponding *p*-value showed that the relationship was not statistically significant, failing to confirm H6.

When deconstructing the total effect into direct and indirect effects via the mediators, both TRUST and SCS were statistically significant in explaining EQ, in turn, confirming H8 and H9, respectively. Each of the predictors of these mediators was statistically significant; thus, the hypotheses H10–H16 were accepted.

### 4.3. The Mediating Effects

Table 5 shows that, after controlling for the first mediator TRUST, the direct effect of the predictors became largely insignificant, except for PAD. The result shows that higher levels of PII, PAN, and HHS developed higher levels of TRUST, which, in turn, resulted in higher levels of EQ. Our result concurs with previous findings that proved the mediating role of the social environment in healthcare settings [72].

Similarly, after controlling for the second mediator, SCS, we found that the direct effect of PIR remained statistically insignificant. In other words, it is not the perceived information reliability per se that shaped EQ, but PIR developed SCS, which, in turn, led to higher levels of EQ. This result also concurs with previous findings, showing that, although patients’ trust in healthcare professionals is not related to their health outcomes, the patients report higher satisfaction when their trust in the professionals is higher [73].

Some other relationships were only partially mediated; the direct effect of PAD on EQ did not lose its significance after extracting the indirect effect via the mediator. The same result held for WTP and ATT; the direct effects of these predictors remained significant after controlling for the mediator.

Table 6 presents the effect sizes of each predictor on the corresponding dependent variable. These values are very useful in deciding which predictor can serve as a potential target for interventions. When lower than 0.02, the effect of the corresponding predictor on the outcome variable is too small to allow for interventions; effects that range between 0.02 and 0.15 are small, those between 0.15 and 0.35 are moderate, and those above 0.35 are strong [74]. Here, the ATT had the highest effect size, 0.279, which can be classified as moderate. SCS was also important, with an effect size of 0.137. Small, but still reasonable candidates for interventions were represented by all the variables whose effect sizes listed in Table 6 were higher than 0.02. The implications of these values are discussed in terms of practical and managerial interventions in the last section of this paper.

**Figure 2:**
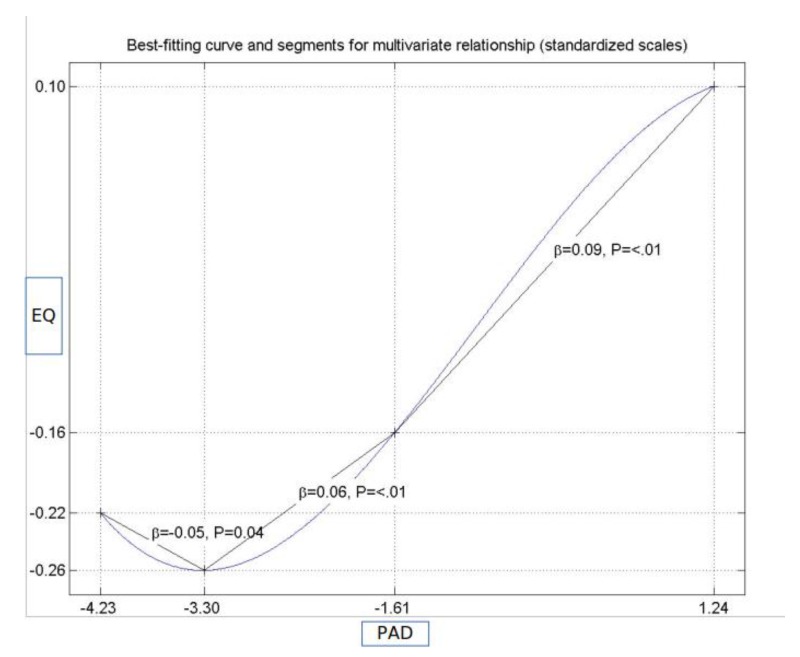
The non-linear relationship between procedural accuracy—doctors (PAD) and patients’ perception of the experienced quality of health services (EQ).

**Figure 3:**
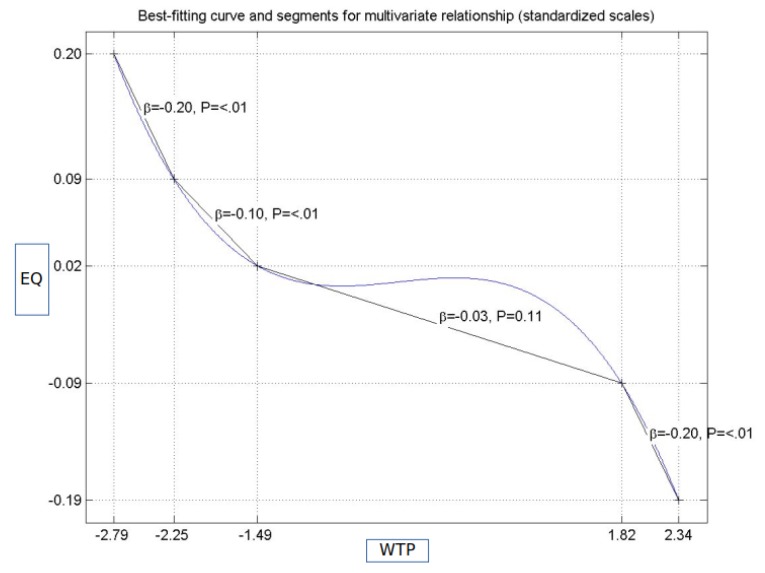
The non-linear relationship between the perception of overall quality and waiting time.

Figure 2 and Figure 3 capture a very important result of our research. While the initial research model assumed linear relationships among variables, we found that two of them were non-linear, including the relationship between PAD and EQ and that between WTP and EQ. Table 5 shows that the relationship between PAD and EQ was positive and statistically significant, in terms of both a direct effect and a total effect. The results imply that, as the score for PAD increased, the score for EQ increased as well, at a constant rate. What Figure 2 shows instead is that the direct relationship between these two variables held only if PAD went above a certain threshold. Moreover, the relationship was barely linear and could be characterized by two different slopes: 0.06 when the score of the perceived doctors’ attitude range was between −3.30 and −1.61 (standardized values), and 0.09 beyond this value. Furthermore, since the curve was convex between −3.30 and −1.61 and concave above −1.61, we expected that the increase in the overall satisfaction was steeper in the first case and slower in the second case.

Similarly, the assumed linear relationship between WTP and EQ was negative. Figure 3 shows, however, that there were three different regions where the negative relationships can be discussed. When the standardized score for WTP was lower than −1.49, the relationship could be described by a decreasing convex function, with a variable slope of −0.20, if the standardized score for WTP ranged between −2.79 and −2.25 (holding also for scores ranging between 1.82 and 2.34, but with a concave shape), and a variable slope of −0.10, for the interval between −2.25 and −1.49. If the standardized score for WTP ranged between −1.49 and 1.82, there was no statistically significant relationship. The implications of the results presented in Figure 2 and Figure 3 are discussed in the section devoted to practical implications (Section 5.1).

## 5. Discussion, Conclusions and Future Research

Our study explored patients’ experience with the quality of the healthcare services in Romania. We considered two categories of predictors. One category was related to human capital, expressed as ATT, WTP, and PIR. The other category was related to social capital and was expressed as the PII between patients and health professionals and health professionals’ procedural accuracy and provided support. We found that TRUST partially mediated the relationship between human capital dimensions and EQ of health services. Similarly, satisfaction by SCS partially mediated the relationship between social capital dimensions and patients’ EQ with healthcare services.

We combined direct and indirect measurements of patients’ satisfaction into a structural equation model, aiming to assess patients’ EQ of health services in Romania. We found that direct assessment was a good predictor for the indirect assessment, thereby confirming the conclusion that the results of these two types of evaluations do not converge [2]. Moreover, we identified two mechanisms through which the relationship holds. Our result may confirm that there is a certain wisdom of patients that eventually makes the measurements consistent [75].

### 5.1. Practical and Managerial Implications

Although using patients’ perspective for improvement is still debated [76], the attempt to use their feedback for simple and practical solutions is an ongoing preoccupation [77]. Given the nature of our results, our recommendations are rather concrete. The effect sizes presented in Table 6 showed that the PAN ranked first in developing trust in patients, then PAD, followed by HHS. Although the effect sizes were small, they were still suitable for practical interventions. We can advance the idea that one of the most efficient solutions to increase patients’ satisfaction and their compliance is to improve nurses’ communication skills. Similar arguments emphasize that reducing information asymmetry through communication with health professionals has a positive impact on patients, which, in turn, increases their satisfaction via trust as a valuable mediator. The implication of this result goes hand in hand with the previous result, pointing toward empowering the communication dimension between patients and health practitioners. Our conclusion aligns with one of the main current research trends, namely, transforming the human side of services [78] and addressing patient’s concerns in a patient-centered way.

One of the most important results regards the second mediator, patients’ satisfaction with health services by specialization. Although many studies emphasize the negative correlation between waiting time and patients’ contentment, we found that the most relevant variable in our case was the degree of attention patients perceive that they received, once the contact with the health practitioner was established. This result suggests that, even with scarce healthcare resources, patients’ satisfaction can be sustained by the quality of attention and care they receive. Our result concurs with other findings [79] and suggests that, by turning waiting time into attention received, patients’ satisfaction can increase. We propose three methods to achieve this goal: (1) informing the patient regarding the reasons for which he/she must wait, shows respect and consideration; (2) by using waiting time to answer specific questions regarding their health problems, the patients can focus on that specific task, rather than on the unpredictable end of the waiting; (3) loyal patients who return to the same medical center can valuably use their waiting time to fill questionnaires regarding their compliance with treatment. Satisfied patients are more adherent to treatment and physician recommendations and more loyal to the respective medical professional and facility. Thus, patient satisfaction contributes to the patient’s experience.

All these suggestions are not only meant to engage the patients as active rather than passive participants, and enhance their involvement, but they are also very useful for the health practitioners. Although patients often miss relevant aspects in their discussion with the doctors, given the time constraints and the stress involved in medical evaluations, additional information would improve the health practitioner’s medical efficiency.

Our results showed that some of the relationships involved in our model were non-linear, as illustrated in Figure 2 and Figure 3. In turn, this suggests that, by both decreasing waiting time and improving the attitude of medical doctors, higher levels of patients’ satisfaction are expected. Conversely, due to non-linearity, the efficiency of these two interventions depends on the initial values of the predictors. Practical interventions aimed at reducing waiting time need to be tailored to ensure that the result falls in the area of relevant intervention (more precisely, scores ranging between −2.79 and −1.49, or between 1.82 and 2.34, as Figure 3 shows). If the intervention ends up with scores between −1.49 and 1.82, it will not have any significant impact on patients’ satisfaction, although it will entail costs. A similar type of reasoning applies to the other non-linear relationships. The most important conclusion derived from our study is that, unless the nature of relationships among the predicting variables and satisfaction is understood, practical interventions could fail to yield positive results. Other studies presented the importance of accounting for non-linear relationships, in particular, when proper interventions should be tailored [80] and provide insights into the advantages of warping over segmentation analysis [81]. Our findings confirm the concerns of other researchers regarding the problems that may arise whenever the relationships among variables are not correctly specified [82].

### 5.2. Limitations and Future Research

Measuring patients’ satisfaction is a complex task, highly dependent on the type of measurement, moment of measurement, type of services, or context [83]. Although our sample was country-representative, our results hold within the limits of the instrument we used and considering that the presence of missing data required imputation procedures. Another important limitation is that, in our attempt to combine direct and indirect measurements, we did not target very specific experiences, but rather overall perceptions. Nevertheless, we see our results as valuable in terms of theoretical contributions and practical and managerial implications.

## Figures and Tables

**Table 1:** Latent variables measurement.

Latent Variable	Acronym	Measurement Items (Likert 1–5)
Perceived intensity of interaction (PII)	To what extent did you discuss with health professionals regarding the following?	
	PII1	The solution to your health issue
	PII2	The treatment/medication for your health issue
Trust (TRUST)	According to your personal experience, rate the level of trust you have in the following categories of health professionals:	
	TRUST_DOCTORS	Doctors
	TRUST_NURSES	Nurses
	TRUST_HH	Hospital housekeepers
Procedural accuracy —doctors (PAD)	PAD1	The doctor acted professionally
	PAD2	The doctor observed confidentiality
	PAD3	The doctor informed you about all possible risks and alternatives related to your treatment
Procedural accuracy —nurses (PAN)	PAN1	The nurse acted professionally
	PAN2	The nurse observed confidentiality
	PAN3	The nurse informed you about all possible risks and alternatives related to your treatment
Hospital housekeepers’ support (HHS)	HHS1	The hospital housekeepers acted professionally
	HHS2	The hospital housekeepers helped you effectively
Waiting time on the premises (WTP)	According to your experience, when confronted with a medical situation, how do you rate the waiting time inside the building in each of the following cases? (1—very short; 5—very long)	
	WTP1	Family physicians
	WTP2	Specialists
	WTP3	Hospital
	WTP4	Laboratory
Satisfaction by category of services (SCS)	How do you rate the quality of the health services you received from the following sources?	
	SCS1	Family physicians
	SCS2	Specialists
	SCS3	Hospital
	SCS4	Laboratory
Attention (ATT)	According to your experience, when confronted with a medical situation, how do you rate the attention you received in each of the following cases?	
	ATT1	Family physicians
	ATT2	Specialists
	ATT3	Hospital
	ATT4	Laboratory
Perceived information reliability (PIR)	To what extent do you trust information from the following sources?	
	PIR1	Family physicians
	PIR2	Specialists

**Table 2:** Descriptive statistics.

Variable	Frequency
Gender	
Female	58.9%
Male	41.1%
Civil status	
Married	61.9%
Divorced	5.8%
Unmarried	14.0%
Consensual union	2.1%
Other	15.9%
Social status	
Similar to other families	61.3%
Above average	15.0%
Among the wealthiest	0.8%
Among the poorest	3.5%
Under average	16.0%
Education	
Maximum 10 years	30.5%
High school	27.5%
Vocational school	26.7%
Bachelor	12.8%
Master	2.4%
Sector	
Public	13.5%
Private	28.2%
Do not work	58.3%
Home place (# inhabitants)	
Village	58.7%
100–200	7.9%
30–100	8.9%
>200	16.9%
<30	7.5%

**Table 3:** Reliability of the measurement.

Variable ^1^	Cronbach’s Alpha (* > 0.7)	Composite Reliability Index (* > 0.7)	Average Variance Extracted (* > 0.5)
PII	0.949	0.975	0.951
TRUST	0.861	0.916	0.784
PED	0.895	0.935	0.827
PAN	0.891	0.932	0.822
HHS	0.935	0.969	0.939
WTP	0.579	0.760	0.446
SCS	0.791	0.865	0.616
ATT	0.819	0.881	0.649
PIR	0.691	0.866	0.764

* Recommended value. ^1^ See Table 1 for definitions of variables.

**Table 4:** Discriminant validity: correlations among latent variables with square roots of AVEs ^1^.

Variable ^2^	PII	TRUST	PAD	PAN	HHS	WTP	SCS	ATT	PIR
PII	0.975	0.388	0.385	0.323	0.223	−0.178	0.440	0.445	0.313
TRUST	0.388	0.885	0.478	0.494	0.393	−0.264	0.520	0.548	0.402
PAD	0.385	0.478	0.909	0.752	0.441	−0.245	0.445	0.637	0.507
PAN	0.323	0.494	0.752	0.906	0.597	−0.253	0.423	0.576	0.425
HHS	0.223	0.393	0.441	0.597	0.969	−0.151	0.270	0.420	0.296
WTP	−0.178	−0.264	−0.245	−0.253	−0.151	0.667	−0.278	−0.345	−0.184
SCS	0.440	0.520	0.445	0.423	0.270	−0.278	0.785	0.584	0.379
ATT	0.445	0.548	0.637	0.576	0.420	−0.345	0.584	0.806	0.505
PIR	0.313	0.402	0.507	0.425	0.296	−0.184	0.379	0.505	0.874

^1^ Average variance extracted. ^2^ See Table 1 for definitions of variables.

**Table 5:** The coefficients of the structural model.

Variable ^1^	Direct Effects			Indirect Effects	Total Effects
	TRUST	Quality by Specialization	Overall Quality	Overall Quality	Overall Quality
TRUST	-	-	0.182 *** (<0.001)	-	0.182 *** (<0.001)
SCS	-	-	0.274 *** (<0.001)	-	0.274 *** (<0.001)
PII	0.226 *** (<0.001)	-	0.039 (0.066)	0.041 * (0.012)	0.080 *** (<0.001)
PAD	0.176 *** (<0.001)	-	0.086 *** (<0.001)	0.032 * (0.039)	0.118 *** (<0.001)
PAN	0.194 *** (<0.001)	-	0.002 (0.463)	0.035 * (0.026)	0.038 (0.072)
HHS	0.150 *** (<0.001)	-	0.024 (0.180)	0.027 (0.067)	0.051 * (0.024)
WTP	-	−0.140 *** (<0.001)	−0.050 * (0.027)	−0.038 * (0.017)	−0.088 *** (<0.001)
ATT	-	0.474 *** (<0.001)	0.075 ** (0.002)	0.130 *** (<0.001)	0.205 *** (<0.001)
PIR	-	0.114 *** (<0.001)	0.016 (0.271)	0.031 * (0.044)	0.047 * (0.034)
Need for medical services	-	-		-	
No			Reference		Reference
Yes			−0.066 ** (0.005)		−0.066 ** (0.005)
AGE	-	-	0.052 * (0.022)	-	0.052 * (0.022)
GENDER:	-	-		-	
Male			Reference		Reference
Female			−0.016 (0.270)		−0.016 (0.270)
*R*^2^/Adjusted *R*^2^	37%	33%	34%	-	-

^1^ See Table 1 for definitions of variables. *** *p* < 0.001; ** *p* < 0.01; * *p* < 0.05; *p* < 0.10.

**Table 6:** Effect sizes of direct effects.

Variable ^1^	Effect Sizes of Direct Effects		
	TRUST	Quality by Specialization	Overall Quality
TRUST	-	-	0.083
SCS	-	-	0.137
PII	0.091	-	0.012
PAD	0.085	-	0.034
PAN	0.096	-	0.001
HHS	0.063	-	0.007
WTP	-	0.050	0.014
ATT	-	0.279	0.033
PIR	-	0.043	0.004
Need for medical services	-	-	0.004
Age	-	-	0.008
Gender	-	-	0.001

^1^ See Table 1 for definitions of variables.

## Supplementary Materials

The following are available online at https://www.mdpi.com/1660-4601/17/1/152/s1, Table S1: Conceptual research model and hypotheses.
