# Supplementary material for: Combining Direct and Indirect Measurements to Assess Patients’ Satisfaction with the Quality of Public Health Services in Romania: Uncovering Structural Mechanisms and Their Implications
Source: Int J Environ Res Public Health. 2019 Dec 24;17(1):152. doi: 10.3390/ijerph17010152 (PMC6981560; doi:10.3390/ijerph17010152)
Supplement: Supplementary file 1 [file ijerph-17-00152-s001.pdf]

	COMUNIC	TRUST HP	ATT DR	ATT NURSES	ATT HH	TIME	QUAL SPEC	ATTENTION	TRUST INFORM
PII1	**0.975**	-0.015	-0.007	0.012	-0.003	-0.003	0.012	0.005	0.004
PII 2	**0.975**	0.015	0.007	-0.012	0.003	0.003	-0.012	-0.005	-0.004
TRUST_DOCTORS	0.078	**0.866**	0.217	-0.165	-0.082	-0.008	-0.023	-0.008	0.044
TRUST_NURSES	-0.007	**0.932**	-0.097	0.188	-0.111	0.008	0.006	0.003	-0.006
TRUST_HH	-0.071	**0.855**	-0.114	-0.038	0.204	-0.001	0.017	0.004	-0.038
PAD1	-0.003	0.066	**0.914**	-0.129	0.026	-0.004	-0.008	0.072	0.012
PAD 2	-0.005	-0.054	**0.906**	0.008	-0.014	0.001	0.025	-0.109	-0.019
PAD 3	0.008	-0.013	**0.908**	0.121	-0.012	0.002	-0.017	0.037	0.007
PAN1	-0.008	0.053	0.021	**0.928**	-0.017	0.002	0.023	0.036	0.006
PAN2	-0.007	-0.036	0.105	**0.916**	-0.087	0.005	0.002	-0.045	-0.002
PAN3	0.016	-0.018	-0.132	**0.874**	0.109	-0.007	-0.026	0.010	-0.004
HHS1	-0.014	0.008	0.010	0.028	**0.969**	0.005	0.016	-0.000	-0.011
HHS2	0.014	-0.008	-0.010	-0.028	**0.969**	-0.005	-0.016	0.000	0.011
WTP1	0.047	0.090	-0.194	0.089	-0.023	**0.604**	-0.018	-0.072	-0.049
WTP2	-0.024	-0.045	0.103	-0.046	0.099	**0.768**	0.004	-0.005	0.000
WTP3	0.029	-0.009	-0.019	0.086	-0.157	**0.703**	-0.039	0.018	0.095
WTP4	-0.052	-0.023	0.088	-0.137	0.083	**0.578**	0.061	0.060	-0.064
SCS1	0.092	-0.084	0.125	-0.039	-0.036	0.055	**0.711**	-0.133	0.211
SCS2	-0.064	0.047	0.102	-0.121	0.013	0.012	**0.848**	0.015	-0.026
SCS3	-0.018	0.030	-0.088	0.018	0.123	-0.038	**0.781**	0.014	-0.154
SCS4	0.003	-0.004	-0.134	0.147	-0.104	-0.025	**0.793**	0.089	-0.010
ATT1	0.016	-0.057	0.327	-0.128	-0.040	0.019	-0.009	**0.765**	0.190
ATT2	0.019	0.058	-0.042	0.017	-0.036	0.036	-0.030	**0.871**	-0.007
ATT3	-0.037	0.020	-0.190	0.076	0.092	-0.007	0.024	**0.794**	-0.115
ATT4	0.001	-0.029	-0.079	0.028	-0.014	-0.051	0.018	**0.789**	-0.061
PIR1	0.012	0.030	0.102	-0.062	-0.029	-0.003	-0.029	0.022	**0.874**
PIR2	-0.012	-0.030	-0.102	0.062	0.029	0.003	0.029	-0.022	**0.874**

Table S1: Combined loadings and cross loadings

	COMUNIC	TRUST	ATTIT DR	ATTIT NURSES	ATTIT HH	TIME	QUAL SPEC	ATTENTION	TRUST INFORM
PII	***0.975***	0.388	0.385	0.323	0.223	-0.178	0.440	0.445	0.313
TRUST	0.388	***0.885***	0.478	0.494	0.393	-0.264	0.520	0.548	0.402
PED	0.385	0.478	***0.909***	0.752	0.441	-0.245	0.445	0.637	0.507
PAN	0.323	0.494	0.752	***0.906***	0.597	-0.253	0.423	0.576	0.425
HHS	0.223	0.393	0.441	0.597	***0.969***	-0.151	0.270	0.420	0.296
WTP	-0.178	-0.264	-0.245	-0.253	-0.151	***0.667***	-0.278	-0.345	-0.184
SCS	0.440	0.520	0.445	0.423	0.270	-0.278	***0.785***	0.584	0.379
ATT	0.445	0.548	0.637	0.576	0.420	-0.345	0.584	***0.806***	0.505
PIR	0.313	0.402	0.507	0.425	0.296	-0.184	0.379	0.505	***0.874***

Table S2. Discriminant validity: Correlations among latent variables with sq. rts. of AVEs
